# Genistein Attenuates Acute Cerebral Ischemic Damage by Inhibiting the NLRP3 Inflammasome in Reproductively Senescent Mice

**DOI:** 10.3389/fnagi.2020.00153

**Published:** 2020-06-17

**Authors:** Shiquan Wang, Jin Wang, Haidong Wei, Tingting Gu, Jiajia Wang, Zhixin Wu, Qianzi Yang

**Affiliations:** ^1^Department of Anesthesiology and Perioperative Medicine, Xijing Hospital, The Fourth Military Medical University, Xi’an, China; ^2^Department of Anesthesiology, The Second Affiliated Hospital of Xi’an Jiaotong University, Xi’an, China

**Keywords:** genistein, cerebral ischemic injury, microglia, NLRP3, reproductively senescent mice

## Abstract

Postmenopausal women have a higher incidence of stroke compared to the age-matched males, and the estrogen was thought to be the main cause of such difference. However, estrogen replacement therapy for the prevention of postmenopausal stroke shows controversial results and is widely disputed because of its serious side effects after chronic administration. Genistein (Gen), a natural phytestrogen with fewer side effects, has a protective effect against cerebral ischemia damage. However, whether Gen could effectively prevent postmenopausal stroke has not been elucidated. In the current study, reproductively senescent mice were treated with Gen (10 mg/kg) for 2 weeks before having transient cerebral ischemia insults. Neurological scores, infarct volumes, and cell apoptosis were evaluated 24 h after reperfusion. The levels of inflammatory factors and nod-like receptor protein 3 (NLRP3) inflammasome-related proteins were also examined. The results showed that Gen treatment reduced infarct volumes, improved neurological scores, attenuated apoptosis, and decreased inflammatory factor release. The expression of NLRP3 inflammasome-related proteins in microglia was downregulated by Gen. However, the overexpression of NLRP3 in microglia abrogated the Gen-induced inhibition of inflammatory factor release and reversed the neuroprotective effect of Gen. Taken together, the results suggest that Gen treatment could attenuate the acute injury induced by cerebral ischemia in reproductively senescent mice *via* the inhibition of the NLRP3 inflammasome in microglia, indicating that Gen could be a candidate drug for the treatment of stroke in postmenopausal women.

## Introduction

Compared with the age-matched men, the incidence of stroke in women is lower in premenopausal period but significantly increases after menopause (Haast et al., 2012). Estrogen is the main cause of the sex differences in stroke incidence. Many animal studies have confirmed that estrogen has a strong neuroprotective function. However, clinical studies have come out with controversial results. Although the hormone therapy could decrease the risk of stroke in postmenopausal women (Henderson and Lobo, 2012), and the treatment with conjugated equine estrogens for 7.2 years was not associated with risk of all-cause, cardiovascular, or cancer mortality during a cumulative follow-up of 18 years (Manson et al., 2017), hormone therapy increases the risk of other undesired consequences, such as invasive breast cancer, intracerebral hemorrhage, and venous thromboembolism (Gartlehner et al., 2017). Fortunately, natural food-derived compounds with structures and functions similar to estrogen could be alternatives to postmenopausal hormone therapy with fewer side effects. As a well-studied plant estrogen, Genistein (Gen) is a kind of isoflavone phytestrogen and has the greatest proportion and accounts for approximately half of total isoflavones found in soy foods. Previous studies showed that Gen could reduce the damages of both focal and global cerebral ischemia (GCI) in overiectomized female mice (Schreihofer and Oppong-Gyebi, 2019). Therefore, we hypothesized that Gen supplementation could potentially be effective in protecting against stroke in the reproductively senescent animals.

Inflammation plays a crucial role in the pathophysiology of ischemic injury. Ischemia-initiated inflammatory responses have various characteristics and are gender-dependent (Dotson and Offner, 2017). This gender difference is mainly attributed to the level of sex hormones, including estrogen, which are important regulators of the inflammatory responses (Bereshchenko et al., 2018). A recently discovered inflammasome, nod-like receptor protein 3 (NLRP3), plays a key role in the mediation of inflammatory responses in ischemic stroke. The activation of the NLRP3 inflammasome aggravates the cerebral damage of stroke, whereas NLRP3 depletion protects mice from cerebral ischemia injury (Gao et al., 2017). However, whether the NLRP3 inflammasome is involved in the neuroprotective effect of Gen against reproductively senescent stroke remains to be confirmed.

In the current study, we intended to determine whether pretreatment with Gen could reduce the damage induced by ischemic stroke in reproductively senescent mice and to explore the underlying role of the NLRP3 inflammasome in the postmenopausal neuroprotection of Gen.

## Materials and Methods

### Animals

Female C57BL/6J mice, 17–18 months old, weighing 28–32 g, were purchased from the Experimental Animal Center of Fourth Military Medical University (Xi’an, China). All animals were housed in an environment with a temperature of 22 ± 2°C, a relative humidity of 50% ± 1%, and a light–dark cycle of 12/12 h. Vaginal smears were obtained from the mice to confirm the cessation of the estrous cycle for seven consecutive days. All animal studies (including the mice euthanasia procedure) were performed in compliance with the regulations and guidelines of Fourth Military Medical University institutional animal care and according to the Accreditation of Laboratory Animal Care International and the Institutional Animal Care and Use Committee guidelines.

### Experimental Design

In the first part of the experiment, to examine the effect of Gen on focal cerebral ischemia in reproductively senescent mice, the animals were randomly divided into four groups: sham, control, Gen, and vehicle groups. All mice except for the sham group were subjected to unilateral middle cerebral artery occlusion (MCAO). In the Gen group, Gen was administered intraperitoneally at a dose of 10 mg/kg, once daily, for 2 weeks, before MCAO as previously described (Wang et al., 2014). Neurological scores, infarct volume, and terminal deoxynucleotidyl transferase-mediated dUTP nick-end labeling (TUNEL) level were analyzed 24 h after reperfusion. Inflammatory cytokines and NLRP3 inflammasome–related proteins were also investigated after ischemia.

To confirm the involvement of microglial NLRP3 in Gen-induced neuroprotection, cultured primary microglia were randomly divided into the following groups: blank (cultured cells with no additional treatment), Gen blank (cultured cells treated with Gen for 24 h), OGD (cells subjected to oxygen glucose deprivation/reperfusion), Gen (cells treated with Gen for 24 h and then subjected to OGD/reperfusion), and vehicle (cells treated with vehicle for 24 h and then subjected to OGD/reperfusion). The expression of NLRP3, pro–caspase-1 and the release of inflammatory cytokines were analyzed.

In the third part, to elucidate the role of microglial NLRP3 in the neuroprotective effect of Gen, cocultured N9 microglia and HT22 cells were randomly assigned to the following treatments: blank (cultured cells received no treatment), OGD (cells subjected to OGD/reperfusion), Gen (N9 microglia cells treated with Gen for 24 h and then cocultured with HT22 cells and subjected to OGD/reperfusion), Gen + NLRP3 (N9 microglia overexpressing NLRP3 were treated with Gen for 24 h and then cocultured with HT22 cells and subjected to OGD/reperfusion), and Gen + control (N9 microglia infected with control virus and then cocultured with HT22 cells and subjected to OGD/reperfusion).

### Drugs Dilution and Treatment

Gen purchased from Selleck Chemicals, Houston, Texas, USA was dissolved in dimethyl sulfoxide and then diluted with saline. For the *in vitro* experiments, the final concentration of Gen was 5 μg/ml. The reproductively senescent mice received intraperitoneal injection of 10 mg/kg Gen or the same volume of vehicle, once per day, for 2 weeks, prior to MCAO (Wang et al., 2014).

### Focal Cerebral Ischemia and Reperfusion

Mice were allowed free access to food and tap water before surgery. Cerebral ischemia was induced by MCAO as previously described (Wang et al., 2014). Briefly, the mice were anesthetized with 1.5% isoflurane. A silicon-coated suture (RWD Life Science, Shenzhen, Guangdong, China) was then inserted into the right external carotid artery and advanced through the internal carotid artery to obstruct the middle cerebral artery. The suture remained in position for 1 h during the arterial occlusion and was then removed to allow subsequent reperfusion. The body temperature of the mice was monitored by a rectal probe and maintained at 37°C ± 0.5°C by using a heating pad. A laser Doppler sensor for blood flow monitoring was placed on the surface of the skull (2 mm caudal and 4 mm lateral to the Bregma). A procedure with 80% decrease and 70% recovery of the regional cerebral blood flow was considered to be a successful ischemic injury. The mice in sham group received the same intervention except that no suture bolt was inserted.

### Assessment of Neurological Deficit

Based on the scoring system described by Garcia et al. (1995), the neurological behavior of mice was assessed 24 h after reperfusion by an observer blinded to the animal groups. The scoring consists of six tests: spontaneous activity, symmetrical movements, symmetry of forelimbs, climbing wall of wire cage, reaction on touch on either side of trunk, and response to vibrissal touch. Each behavior was ranked based on a scale between 0 and 3 points, and a total score was the sum of all six individual tests. In the first three tests, behaviors were classified into no movement (0), slight movement (1), slow movement (2), and normal movement (3). In the last three tests, behaviors were classified into no movement or response (1), weak movement or response (2), and normal movement or response (3). The higher scores represented better neurological outcomes.

### Measurement of Infarct Size

After the mice were euthanized, the brains were removed. The brains were first sectioned into 1-mm slices. Then, the sections were incubated in a 2% solution of 2,3,5-triphenyltertrazolium chloride at 37°C for 15 min and fixed in 4% formalin. The stained sections were photographed using a digital camera and measured in a blinded manner with image analysis software. In consideration of tissue edema, the ratio of infarct volume was calculated according to the following equation: infarct ratio = (contralateral hemispheres − noninfarcted areas of ipsilateral hemispheres)/contralateral hemispheres (Wang et al., 2014).

### OGD/Reperfusion

Briefly, the cell culture medium was changed to Dulbecco modified eagle medium (DMEM) without glucose, glutamine, and sodium pyruvate (Gibco, Grand Island, New York, USA), and the cells were transferred to a modular incubator chamber and flushed with 3 l/min of a 95% N_2_ and 5% CO_2_ gas mixture for 15 min at room temperature. The chamber was then sealed and placed in a 37°C container. OGD was carried out for 2 h, and then the cells were incubated with normal growth medium for an additional 12 h of reperfusion under normal conditions (Liu et al., 2018).

### Cell Viability Assay

A cell counting kit 8 (CCK-8; 7Sea Biotech, Shanghai, China) was used to assess cell survival according to the manufacturer’s instructions. Briefly, 50 μl of CCK-8 solution was added into 500 μl of medium solution in each culture well of a 24-well plate and incubated for 4 h at 37°C. The absorbance at 450 nm was measured with a microplate reader (Infinite M200; TECAN, Switzerland).

### Lactate Dehydrogenase Release Assay

The lactate dehydrogenase (LDH) cytotoxicity colorimetric assay kit (K313-500; Biovision, San Francisco, California, USA) was used to detect cell injury. The assessment was performed according to the manufacturer’s instructions. Briefly, medium (50 μl per well) from each cell culture well of a 24-well plate was added to an optically clear 96-well plate. Then 50 μl of LDH reaction mix was added to each well, mixed, and incubated for 30 min at room temperature. The absorbance at 490 nm (in reference to 690 nm) was measured with a microplate reader (Infinite M200; TECAN, Switzerland).

### TUNEL Staining

Cellular apoptosis was evaluated at 24 h after reperfusion. TUNEL staining was performed using an *in situ* cell death detection kit (Roche Diagnostics, Mannheim, Germany) according to the manufacturer’s instructions. Mice brains were fixed with 4% Paraformaldehyde. The tissue was then cut into 12-μm-thick coronal sections from 0.5 mm prior to Bregma. Three slices for each mice were used for TUNEL staining. Three fields from the penumbra zone for each slice were observed using a 40× objective lens. The ratio of TUNEL and NeuN double-positive cells to NeuN-positive cells was considered the apoptosis index. The ischemic penumbra area was defined as previously described (Ashwal et al., 1998). Briefly, the brain was sectioned into three slices: section 1 was 2 mm from the anterior tip of the frontal lobe, section 2 was 4 mm, and section 3 was 2 mm. Section 2 that corresponded to the ischemic core and penumbra was dissected. The midline between the two hemispheres was identified, and a longitudinal cut (from top to bottom) approximately 1 mm from the midline through infarct hemisphere was made. We then made a transverse diagonal cut at approximately the 2-o’clock position to separate the core (i.e., striatum and overlying cortex) from the penumbra (adjacent cortex).

### Primary Microglia Culture and Neuron–Microglia Coculture

Primary mouse microglia cultures were harvested from 1- to 2- day-old neonatal C57BL/6J pups. Briefly, the cortical tissues were subjected to enzymatic digestion and mechanical isolation. The mixed cortical cells were then passed through a 70-μm nylon mesh cell strainer and seeded into a cell culture flask in DMEM containing 10% FBS (Gibco) and 1% penicillin/streptomycin. Seven days later, the mixed glial cultures were shaken on an orbital shaker at 200 revolutions/min (rpm) for 2 h. Then, the detached microglial cells in the supernatant were collected and reseeded into cell culture containers. The purity of the microglia in culture was more than 95% as confirmed by staining with the microglia marker Iba-1.

For the indirect neuron–microglia coculture, neurons were seeded in 24-well plates and incubated for 10 days. Primary microglia (microglia: neurons = 1:2) were added to Transwell inserts with 0.4-μm pores (Costar, Shanghai, China) for 3 days. Then, the cocultured neurons and microglia were subjected to different treatments.

### N9-HT22 Coculture

For the indirect N9-HT22 coculture, HT22 cells were seeded in 24-well plates, and N9 microglia were added to Transwell inserts with 0.40 μm pores (Costar, USA). After incubation for 3 days, the cocultured N9 microglia and HT22 cells received the subsequent treatments.

### Immunofluorescence Staining

Immunofluorescence staining was performed on frozen coronal sections of mouse brains or on cultured cells. The mouse brains were fixed with 4% paraformaldehyde. After fixation and concentration gradient dehydration, the brains were cut into 12-μm-thick sections. The cultured cells were fixed with 4% paraformaldehyde for 10 min. The brain sections and cell cover slips were washed three times with phosphate-buffered saline (PBS) and then incubated with primary antibodies overnight at 4°C in a humidified atmosphere. The following primary antibodies were used: mouse anti-NLRP3 (1:100; Adipogen, San Diego, California, USA), rabbit anti-NeuN (1:300; Millipore, Massachusetts, USA), goat anti–Iba-1 (1:200; Abcam, Cambridge, London, UK), and chicken anti–MAP-2 (1:200; Abcam, Cambridge, London, UK). Then, the samples were incubated with a mixture of Alexa-488–conjugated donkey anti-mouse (1:300; Abcam, Cambridge, London, UK), Alexa-594-conjugated donkey anti-rabbit (1:300; Abcam, Cambridge, London, UK), Alexa-594–conjugated donkey anti-chicken (1:300; Jackson ImmunoResearch, Pennsylvania, PA, USA), and Alexa-405–conjugated donkey (1:300; Jackson ImmunoResearch, Pennsylvania, PA, USA) anti-goat secondary antibodies for 2 h in the dark at room temperature. Finally, the sections were photographed using an Olympus BX51 (Tokyo, Japan) fluorescence microscope.

### Western Blotting

Penumbra was dissected from brain ischemia and then homogenized in the RIPA lysis buffer (Beyotime, Nantong, China) containing a whole proteinase inhibitor cocktail. A BCA protein assay kit (Beyotime) was used to determine the protein concentration. The extracted proteins were separated by 10% sodium dodecyl sulfate–polyacrylamide gel electrophoresis and electrically transferred to polyvinylidene difluoride membranes. Then, the membranes were blocked with 5% nonfat milk for 1 h at room temperature. The following primary antibodies were used: mouse anti-NLRP3 (1:1,000; Adipogen, San Diego, California, USA), rabbit anti-pro–caspase-1 (1:1,000; Santa Cruz, California, USA), rabbit anti-cleaved–caspase-1 (1:3,000; Adipogen, San Diego, California, USA), goat anti–Iba-1 (1:2,000; Abcam, Cambridge, London, UK), and mouse anti-GAPDH (1:1,000; Cell Signaling Technology, Boston, Massachusetts, USA). The membranes were shaken at 60 rpm at 4°C overnight and incubated with a secondary anti-rabbit or mouse antibodies (1:10,000; Thermo Scientific, Massachusetts, USA) for 2 h at room temperature. The protein bands were visualized using Bio-Rad system.

### Evaluation of Inflammatory Factors

The penumbra tissue was homogenized in cold normal saline after dissociated and weighted. The homogenate was centrifuged at 10,000 *g* for 15 min, and the supernatant was collected and frozen at −80°C for later detection. In order to test the release of inflammatory factors in cultured cells, the culture medium was collected and frozen at −80°C for later detection. Enzyme linked immunosorbent assay kits (Nanjing Jianchen Bioengineering Institute, Nanjing, Jiangsu, China) were used to assess the content of inflammatory factors [tumor necrosis factor α (TNF-α), interleukin 1β (IL-1β), IL-6, IL-18, and cleaved caspase-1] in strict accordance with the manufacturer’s protocols.

### Lentivirus Transfection

The lentivirus for NLRP3overexpression and the control lentivirus was obtained commercially from Genechem Company (Shanghai, China). The component sequence was as follows: ubi-MCS-3FLAG-SV40-EGFP-IRES-puromycin. N9 microglia were seeded in a 25-cm^2^ culture flask and transfected continuously with LV-NLRP3 or LV control for 12 h. Subsequently, the medium was replaced with fresh medium. The gene overexpression of NLRP3 in N9 microglia was verified using Western blotting. N9 microglia infected with lentivirus were collected and frozen after the medium was changed.

### Flow Cytometric Analysis of Cell Apoptosis

The apoptotic index was detected by flow cytometry with an apoptosis detection kit (7Sea Biotech). Briefly, following the corresponding treatments, the cells were digested with 0.25% trypsin, collected from 6-well culture plates, washed twice with PBS, and centrifuged at 3,000 *g* at 4°C for 10 min. Then, the cells were incubated with 5 μl of fluorescein isothiocyanate–conjugated annexin V dye at room temperature for 15 min, which was followed by 10 μl of propidium iodide dye for 5 min in the dark. Finally, the cells were analyzed by fluorescence-activated cell sorting *via* a flow cytometric analysis (Bio-Rad Laboratories, Inc., Hercules, CA, USA) and CytExpert 1.0 software (Beckman Coulter, Inc., Brea, CA, USA).

### Statistical Analysis

Statistical analyses were performed using SPSS (version 19.0; IBM Corp, Armonk, NY, USA). All data except for neurological scores were presented as the mean with standard deviation (mean ± SD). Multiple comparisons of infarct volume were conducted with one-way analysis of variance (ANOVA), followed by Tukey *post hoc* test. Neurological scores were presented as medians with ranges. The multiple comparisons of neurological scores and other biological tests with small samples (*n* = 4) were analyzed by the Kruskal–Wallis test followed by Dunn test. *P* < 0.05 was considered statistically different.

## Results

### Gen Treatment Alleviated Cerebral Ischemic Injury in Reproductively Senescent Mice

**Figure 1:**
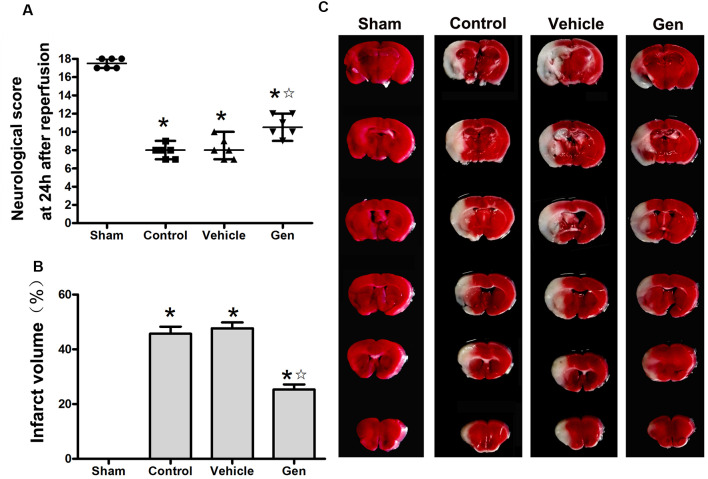
Genistein pretreatment exerted neuroprotective effect against cerebral ischemia injury in reproductively senescent mice. **(A)** The neurological deficit scores evaluated 24 h after reperfusion in reproductively senescent mice after middle cerebral artery occlusion (MCAO). The data are presented as the median with range and analyzed by the Kruskal–Wallis test followed by Dunn test. **(B)** The infarct volumes as percentages of the contralateral hemisphere are presented as the mean ± SD and analyzed by one-way analysis of variance (ANOVA) with Tukey *post hoc* test. **(C)** Representative photographs of brain slices showing infract volume assessed 24 h after reperfusion in reproductively senescent mice. *P < 0.05 compared to the sham group, 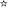
*P* < 0.05 compared to the vehicle group, *n* = 6 per group.

The effect of Gen administration on infarct volume and neurological deficit in reproductively senescent animals 24 h after cerebral ischemia was observed. As shown in Figure 1A, there was no significant difference between the vehicle group [7.5 (1.5)] and control group [8.5 (2)]. Treatment with Gen induced an increase in neurological scores [11 (2; *P* < 0.05 vs. vehicle)]. As shown in Figure 1B, there was no significant difference of infarct volume between the vehicle group and control group (46.8% ± 5.7% vs. 45.6% ± 6.4%, *P* > 0.05).Compared with the vehicle group, Gen pretreatment decreased the infarct size (25.3% ± 4.6%, *P* < 0.05). Representative photomicrographs of cerebral infarct are shown in Figure 1C.

**Figure 2:**
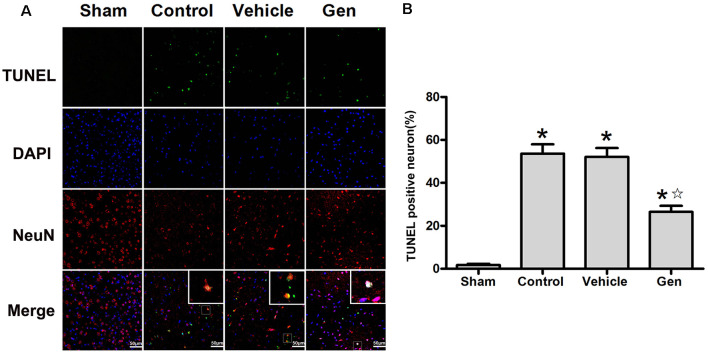
Genistein pretreatment alleviated neuronal injury in the ischemic penumbra. **(A)** Representative photomicrographs showing TUNEL staining in the ischemic penumbra of reproductively senescent mice at 24 h after reperfusion. **(B)** The percentages of TUNEL-positive cells in the ischemic penumbra. Data are presented as the mean ± SD and analyzed by the Kruskal–Wallis test followed by Dunn test. *P < 0.05 compared to the sham group, 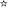
*P* < 0.05 compared to the vehicle group, *n* = 4.

The apoptosis indicated by TUNEL was assessed 24 h after stroke. Representative photomicrographs of TUNEL staining in the ischemic penumbra area are shown in Figure 2A. Treatment with Gen decreased the number of TUNEL-positive neurons compared to the vehicle groups (30.5% ± 7.5% vs. 53.6% ± 9.2%, *P* < 0.05). No significant difference in the number of TUNEL-positive neurons was observed between the control (52.1% ± 9.2%) and vehicle groups (*P* > 0.05), as shown in Figure 2B.

### Gen Pretreatment Reduced Both the Inflammatory Response and Microglial Expression of the NLRP3 Inflammasome in the Cerebral Ischemia Penumbra

**Figure 3:**
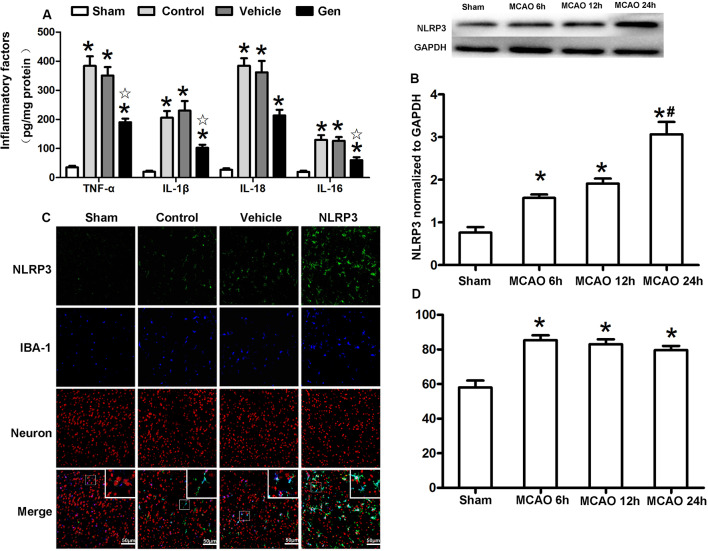
Cellular localization and the time effect of NLRP3 after MCAO in cerebral ischemia penumbra. **(A)** The release of inflammatory factors in the ischemic penumbra of reproductively senescent mice at 24 h after reperfusion. **(B)** Western blotting analysis of the NLRP3 protein expression at different time points after cerebral ischemia. **(C)** The expression and localization of NLRP3 in microglia and neurons at 6, 12, and 24 h after reperfusion. **(D)** The percentages of NLRP3 colocalized with microglia in ischemia penumbra at different time points after cerebral ischemia. Data are presented as the mean ± SD and analyzed by the Kruskal–Wallis test followed by Dunn test. *P < 0.05 compared to the sham group, 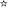
*P* < 0.05 compared to the vehicle group, ^#^*P* < 0.05 compared to the group of MCAO 12 h, *n* = 4 per group.

To verify the anti-inflammatory function of Gen after reproductively senescent stroke, we evaluated inflammatory factors, including TNF-α, IL-1β, IL-18, and IL-6 in ischemic penumbra area 24 h after reperfusion (Figure 3A). Compared with the vehicle group, Gen treatment reduced all these inflammatory factors: TNF-α (190.2 ± 28.0 vs. 383.9 ± 73.5 pg/mg, *P* < 0.05), IL-1β (102.2 ± 24.1 vs. 206.2 ± 51.2 pg/mg, *P* < 0.05), IL-18 (213.48 ± 43.7 vs. 384.2 ± 58.4 pg/mg, *P* < 0.05), and IL-6 (60.0 ± 22.4 vs. 129.9 ± 35.8 pg/mg, *P* < 0.05). There was no differences between the vehicle group and control group: TNF-α (290.4 ± 38.3 vs. 383.9 ± 73.5 pg/mg, *P* > 0.05), IL-1β (203.5 ± 26.3 vs. 206.2 ± 51.2 pg/mg, *P* > 0.05), IL-18 (364.8 ± 33.6 vs. 384.2 ± 58.4 pg/mg, *P* > 0.05), and IL-6 (110.0 ± 28.6 vs. 129.9 ± 35.8 pg/mg, *P* > 0.05).

The expression and cellular localization of the NLRP3 inflammasome were detected within 24 h after reperfusion (Figures 3B–D). The protein expression of NLRP3 began to increase at 6 h after MCAO (1.58 ± 0.14 vs. 0.76 ± 0.22, *P* < 0.05) and was further increased at 24 h (3.06 ± 0.5 vs. 1.91 ± 0.21, 24-h group vs. 12-h group, *P* < 0.05, Figure 3B). Although NLRP3 gradually increased over the 24 h after MCAO, NLRP3 was continuously colocalized with Iba-1–positive microglia (>80%) during this period (Figures 3C,D), indicating that NLRP3 was primarily activated in the microglia after stroke.

**Figure 4:**
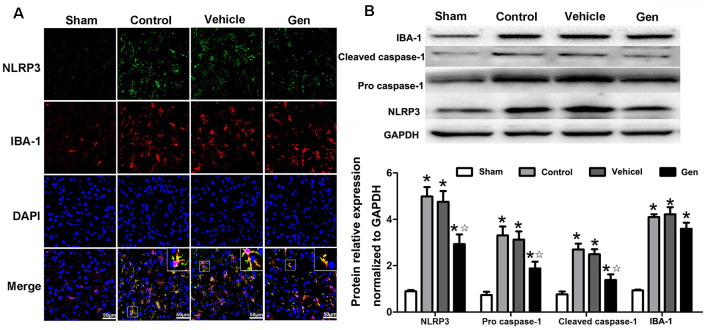
The effect of Gen on expressions of NLRP3 inflammasome related proteins. **(A)** Representative photomicrographs of NLRP3 staining in the ischemic penumbra. **(B)** Western blotting analysis of the related proteins’ expressions at 24 h after reperfusion. Data are presented as the mean ± SD and analyzed by the Kruskal–Wallis test followed by Dunn test. *P < 0.05 compared to the sham group, 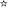
*P* < 0.05 compared to the vehicle group, *n* = 4 per group.

NLRP3 expression in microglia was significantly reduced by Gen pretreatment 24 h after reperfusion (Figure 4A). Pro–caspase-1 and cleaved–caspase-1 expression represents the activity of NLRP3 inflammasome. As shown in Figure 4B, compared with the vehicle treatment, Gen treatment decreased the expression of NLRP3 (2.93 ± 0.70 vs. 4.99 ± 0.70, *P* < 0.05), pro–caspase-1 (1.88 ± 0.50 vs. 3.03 ± 0.67, *P* < 0.05), and cleave–caspase-1 (1.39 ± 0.41 vs. 3.23 ± 0.45, *P* < 0.05). The vehicle had no effect on the levels of these proteins: NLRP3 (4.33 ± 0.708 vs. 4.99 ± 0.70, *P* > 0.05), pro–caspase-1 (2.88 ± 0.53 vs. 3.03 ± 0.67, *P* > 0.05), and cleave–caspase-1 (3.41 ± 0.51 vs. 3.23 ± 0.45, *P* > 0.05). Notably, Gen did not reduce the activation of microglia, as the IBA-1 in penumbra zone in Gen group was not different from control (Figure 4B).

### Gen Reduced the Expression and Activity of the NLRP3 Inflammasome in Microglia After OGD/Reperfusion

**Figure 5:**
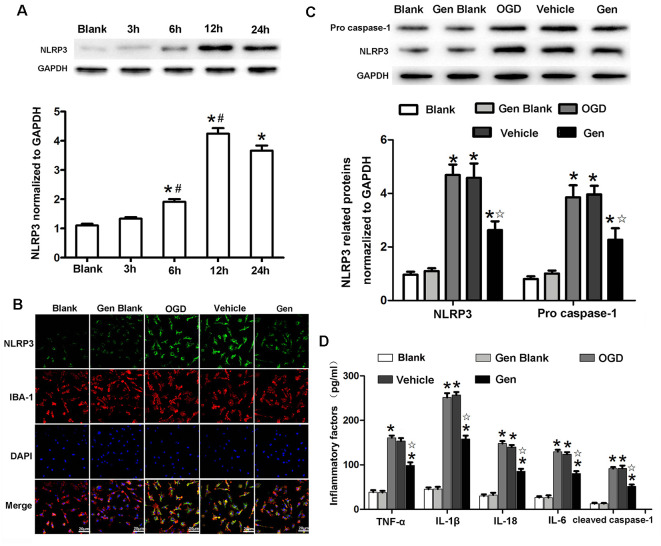
Genistein reduced the expression of NLRP3 inflammasome related proteins in microglia after OGD/R. **(A)** Western blotting analysis of the NLRP3 protein expression at different time points after OGD/R. **(B)** Representative photomicrographs showing NLRP3 staining in microglia at 12 h after OGD/R. **(C)** Western blotting analysis of the inflammasome related proteins’ expressions at 12 h after reperfusion. **(D)** The effect of Gen on the expression of inflammatory factors at 12 h after OGD/R. Data are presented as the mean ± SD and analyzed by the Kruskal–Wallis test followed by Dunn test. *P < 0.05 compared to the sham group, 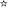
*P* < 0.05 compared to the vehicle group, ^#^*P* < 0.05 compared to previous group, *n* = 4 per group.

Because NLRP3 was mainly expressed in the microglia 24 h after MCAO *in vivo*, we further investigated the specific involvement of microglial NLRP3 in Gen-induced neuroprotection by using an *in vitro* OGD model. As shown in Figure 5A, the expression of NLRP3 began to increase at 6 h (1.91 ± 0.15 vs. 1.33 ± 0.10, 6 h vs. 3 h, *P* < 0.05) and reached its peak at 12 h (3.84 ± 0.32 vs. 1.91 ± 0.15, *P* < 0.05) after OGD/reperfusion. The expression of NLRP3 showed no significant change between 12 and 24 h. In cultured cells, because the expression of NLRP3 remained unchanged between 12 and 24 h after OGD/reperfusion, we chose 12 h as the observation time point for the comparison of inflammasome activity *in vitro*. The expression of NLRP3 at 12 h in microglia after OGD/reperfusion was assessed by immunofluorescence and Western blotting as shown in Figures 5B,C. NLRP3 (2.62 ± 0.57 vs. 4.68 ± 0.67, *P* < 0.05) and pro–caspase-1 (2.27 ± 0.74 vs. 3.85 ± 0.77, *P* < 0.05) were decreased in the Gen-treated group compared to the vehicle group; there was no difference between the control group and vehicle group: NLRP3 (4.42 ± 0.53 vs. 4.68 ± 0.67, *P* < 0.05) and pro–caspase-1 (3.27 ± 0.84 vs. 3.85 ± 0.77, *P* < 0.05). As shown in Figure 5D, the inflammatory factors and cleaved–caspase-1 were decreased in Gen group compared with the vehicle group: TNF-α (98.7 ± 13.7 vs. 153.3 ± 13.7, *P* < 0.05), IL-1β (157.9 ± 15.6 vs. 256.9 ± 13.4, *P* < 0.05), IL-18 (80.3 ± 11.1 vs. 123.7 ± 10.1, *P* < 0.05), IL-6 (85.7 ± 10.8 vs. 139.8 ± 9.5, *P* < 0.05), and cleaved–caspase-1 (51.7 ± 8.3 vs. 92.1 ± 12.5, *P* < 0.05); there was no difference between control group and vehicle group. We also measured the effects of Gen-pretreated microglia on neuronal damage after OGD and found that pretreating microglia with Gen could reduce the degree of neuronal damage after OGD in neuron–microglia coculture system (Supplementary Figure S1).

### NLRP3 Overexpression Reversed the Protective Effect of Gen

**Figure 6:**
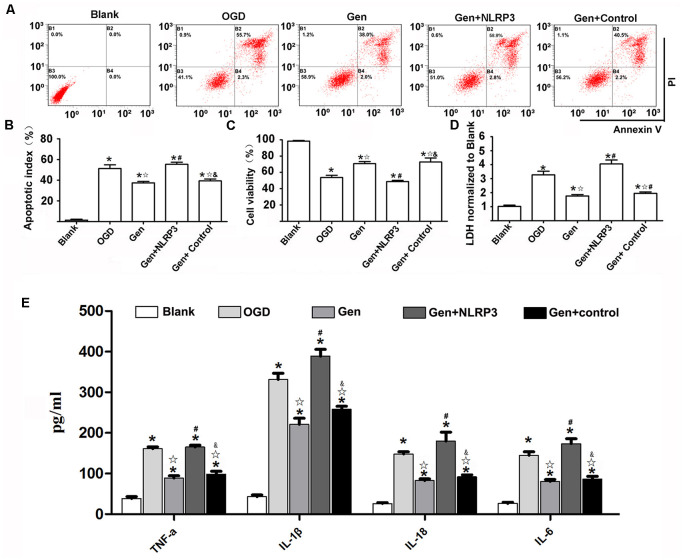
NLRP3 overexpression in microglia partially reversed the protective effect of Gen. **(A)** The representative apoptosis cytometry analysis of HT22 in N9 microglia–HT22–coculture system after OGD/R. **(B)** The apoptosis analysis of HT22 in different groups at 12 h after OGD/R. **(C)** The cell viability of HT22 in different groups at 12 h after OGD/R. **(D)** The release of lactate dehydrogenase (LDH) in different groups at 12 h after OGD/R. **(E)** The release of inflammatory factors at 12 h after OGD/R. Data are presented as the mean ± SD and analyzed by the Kruskal–Wallis test followed by Dunn test. *P < 0.05 compared to the sham group, 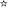
*P* < 0.05 compared to OGD group, ^#^*P* < 0.05 compared to Gen group, ^&^*P* < 0.05 compared to Gen + NLRP3 group, *n* = 4 per group.

To determine the role of the NLRP3 inflammasome in Gen-induced neuroprotection, we overexpressed NLRP3 with lentivirus in N9 microglia in an HT22-N9 cell coculture system. In each group, NLRP3 expression in N9 cells in the coculture system was examined after OGD (Supplementary Figure S2). The HT22 cell apoptosis, cell viability, LDH release, and inflammatory factor expression were observed after OGD/reperfusion is shown in Figure 6. Representative cytometry analysis of HT22 cells apoptosis after OGD/reperfusion was shown in Figure 6A. The quantification of apoptotic HT22 cells was illustrated in Figure 6B. Compared to OGD group, Gen pretreatment reduced HT22 cells apoptosis (37.43% ± 2.42%, *P* < 0.05). Although the overexpression of NLRP3 in N9 microglia abrogated the Gen-induced decrease in neuronal apoptosis (48.46% ± 5.24% *P* < 0.05), the control virus had no effects on neuronal apoptosis (*P* > 0.05). The cell viability and LDH release of HT22 cells were also measured. Compared to OGD group, Gen pretreatment increased cell viability (70.73% ± 3.57% vs. 53.70% ± 3.87%, *P* < 0.05) and decreased LDH release (1.77 ± 0.19 vs. 3.27 ± 0.37, *P* < 0.05). In contrast, the overexpression of NLRP3 in N9 microglia completely counteracted the Gen-induced increase in cell viability (48.66% ± 2.13% vs. 70.73% ±3.57%, *P* < 0.05) and reduction in LDH release (4.05 ± 0.40 vs. 1.77 ± 0.18, *P* < 0.05), whereas the control virus had no effects on cell viability (72.66% ±7.13% vs. 70.73% ± 3.57%, *P* > 0.05) or LDH release (1.95 ± 0.14 vs. 1.77 ± 0.18, *P* > 0.05).

Figure 6E presents the inflammatory factor release results; the inflammatory factors were decreased in the Gen group compared to the OGD group: TNF-α (88.80 ± 11.10 vs. 160.92 ± 9.81 pg/mg, *P* < 0.05), IL-1β (220.86 ± 30.34 vs. 331.34 ± 31.42 pg/mg, *P* < 0.05), IL-18 (82.6 ± 8.19 vs. 147.96 ± 11.04 pg/mg, *P* < 0.05), and IL-6 (80.66 ± 9.51 vs. 144.98 ± 16.83 pg/mg, *P* < 0.05). However, the overexpression of NLRP3 in N9 microglia counteracted the Gen-induced decrease in inflammatory factors: TNF-α (165.32 ± 9.19 vs. 88.80 ± 11.10 pg/mg, *P* < 0.05), IL-1β (220.86 ± 30.34 vs. 331.34 ± 31.42 pg/mg, *P* < 0.05), IL-18 (179.84 ± 43.72 vs. 82.60 ± 8.19 pg/mg, *P* < 0.05), and IL-6 (173.7 ± 24.2 vs. 80.66 ± 9.51 pg/mg, *P* < 0.05), while compared with Gen treatment alone, the control virus had no effect on these inflammatory factors.

## Discussion

The current study demonstrated that the administration of Gen in the reproductively senescent mice could reduce the infarct volume, improve the neurological scores, decrease the neuronal apoptosis, and reduce the release of inflammatory factor release. In addition, Gen also inhibited the NLRP3 inflammasome, as evidenced by the decrease in protein expression of NLRP3, pro–caspase-1, and cleaved caspase-1 in microglia. By using a cell coculture system, Gen-induced neuroprotection against OGD/reperfusion injury was found to be associated with the inhibition of the microglial NLRP3 inflammasome. These results indicate that Gen administration is a preventive approach for stroke in the reproductively senescent mice, and its neuroprotection mechanism involves microglial NLRP3 inflammasome inhibition.

Estrogen decline is considered the key causation of increased risks of postmenopausal stroke. Many studies have documented the neuroprotective effects of estrogen in ischemic stroke (Shao et al., 2012). Then, estrogen replacement therapy, consisting of estrogen alone, or in combination with a progestogen could have been an ideal method for the treatment and prevention of postmenopausal stroke (Grodstein et al., 1996). However, clinical evidences come out with controversial results (Sohrabji et al., 2019). Some researchers found that estrogen replacement therapy has no effect on stroke, and some even reported the increased risk of ischemia. The variance of dosage, administration approach, timing, and patient age could be the contributors to the difference of results. The controversial results and discrepancy between animal studies and clinical data emphasize the importance of performing further investigations using appropriate animal models, modification of estrogen-based therapy, and gaining a deeper understanding of the mechanisms of estrogen-mediated neuroprotection (Manson et al., 2017). Phytestrogen may shed a light on estrogen-based method for postmenopausal stroke, as some natural compounds sharing structural and functional similarities with steroid hormones, particularly estrogens, may have fewer side effects than does estrogen in the prevention of postmenopausal stroke. Genistein is one of the most studied natural compounds. Substantial amounts of Gen are found in soybeans or soy foods such as tofu and soymilk. Interestingly, the frequent intake of foods containing high levels of Gen is associated with a reduced risk of stroke (Kokubo et al., 2007; Liang et al., 2009). As Gen could pass through the blood–brain barrier with few toxic effects (Ganai and Farooqi, 2015) and was also reported to protect rodents from both focal and GCI in male or ovariectomized female animals (Donzelli et al., 2010; Wang et al., 2013, 2014), the use of Gen for the prevention of postmenopausal stroke is promising. Fortunately, in the current study, we found that Gen pretreatment improved neurological outcome, reduced infarct volume, and decreased cellular apoptosis in naturally reproductively senescent mice, suggesting its preclinical potential in postmenopausal stroke prevention.

We found that Gen treatment could potently inhibit the release of inflammatory factors both in MCAO model and OGD model as described by the previous reports (Schreihofer and Oppong-Gyebi, 2019). As Gen is able to bind to estrogen receptors (ERs) and mimic the effect of estrogen, three ERs, ERα, ERβ (Cooke et al., 2006), and G protein–coupled ER (GPR30; Maggiolini et al., 2004), are all involved in the actions of Gen. Moreover, the activation of these receptors could inhibit inflammatory responses (Vegeto et al., 2008), which plays a pivotal role in the ischemia/reperfusion (I/R) injury (Dziedzic, 2015). In addition to its actions on ERs, Gen also interacts with several other receptors in neuroprotection. Genistein could activate the aryl hydrocarbon receptor, which negatively regulates NLRP3 inflammasome activity by inhibiting NLRP3 transcription (Huai et al., 2014; Bialesova et al., 2015). Interleukin R4, which activates NLRP3 inflammasome, could be inhibited by Gen. The anti-inflammatory effects of Gen could also be attributed to the activation of Peroxisome proliferator–activated receptor γ (Dang et al., 2003). Given the extensive actions of Gen, although it is no easy to deduce which receptor or pathway was involved in the anti-inflammatory effect of Gen pretreatment on reproductively senescent mice, the NLRP3 inflammasome took an important part as the results demonstrated.

The NLRP3 inflammasome is the key mediator of inflammatory responses and cellular damage after I/R injury in different organs (Guo et al., 2016). Under immune challenge, the NLRP3 protein is activated and interacts with ASC and pro–caspase-1 to form the NLRP3 inflammasome. The NLRP3 inflammasome induces the transformation of the pro–caspase-1 to caspase-1, catalyzing the formation of mature IL-1β and IL-18 (Sutterwala et al., 2014). More importantly, the activation of the NLRP3 inflammasome aggravates ischemic stroke injury, whereas NLRP3 depletion reduces brain damage after cerebral ischemia (Guo et al., 2016; Alishahi et al., 2019). Therefore, the modulation of NLRP3 inflammasome activation at the molecular level may give us an insight into the development of new therapeutics for ischemic stroke. However, the cell-specific expression of NLRP3 was not well understood, as diverse reports have previously indicated different cell-specific expression of NLRP3 after experimental stroke (Fann et al., 2013; Yang et al., 2014; Gustin et al., 2015). NLRP3 was mainly reported to express in microglia after both ischemic stroke and hemorrhagic stroke (Lu et al., 2016; Ye et al., 2017; Luo et al., 2019). There were some studies indicating that NLRP3 was also increased in endothelia cells after MCAO in the acute phase (Yang et al., 2014). However, the expression of NLRP3 in neurons after cerebral ischemia is probably chronological. Some studies reported the neuronal increase of NLRP3 at the third day after stroke (Jiang et al., 2019), but some demonstrated no expression within 24 h (Zuloaga et al., 2015). Whether there is NLRP3 in astrocytes after stroke is still questionable, although very few *in vitro* studies suggested the NLRP3 increased in astrocytes, under lipopolysaccharide stimulation (Alfonso-Loeches et al., 2014). In the current study, we found greater than 80% of NLRP3 was expressed in the microglia within 24 h after MCAO in the reproductively senescent mice, we subsequently chose the microglia as the target of this study. But, we cannot exclude the possible roles of NLRP3 in neurons and endothelia cells, because we observed only very few colocalization of NLRP3 with neurons (Figure 3C) and slight expression of NLRP3 in astrocytes (data not shown).

A recent study revealed that the activated NLRP3 inflammasome was first formed in microglia 6 h after cerebral injury and was subsequently activated in neurons and vascular endothelial cells in the ischemic core at 24 h (Gong et al., 2018). This indicates that the NLRP3 inflammasome is dynamically regulated after brain damage. Regarding the dynamic change in NLRP3 after stroke in reproductively senescent mice, we found a gradual increase in NLRP3 over 24 h in microglia after brain ischemia. This result is different from the previous ones, and this difference might be attributed to the sex, age (the most important factor) of the animals, and the sampling location. More interestingly, we found that Gen inhibited the NLRP3 inflammasome in reproductively senescent mice 24 h after reperfusion. The inhibitory effect of Gen on NLRP3 after brain ischemia injury has rarely been reported, particularly in postmenopausal stroke.

It has been reported that the suppression of NLRP3 inflammasome in microglia could protect neurons from inflammatory damage (Xu et al., 2018). To further verify the role of microglia NLRP3 in Gen-induced neuroprotection, we performed *in vitro* experiments in a neuron–microglia coculture system. In microglia, we found that the level of NLPP3 was increased at 6 h after OGD/reperfusion and reached the maximum level at 12 h after OGD/reperfusion. This finding is consistent with other reports (Qiu et al., 2016). We selected 12 h after OGD/reperfusion as the subsequent research time point for cell cultures. Similar to animal study, we found that Gen could inhibit the NLRP3 inflammasome in microglia after OGD/reperfusion. We also confirmed that Gen pretreatment exerted a neuroprotective effect on the neurons in the coculture system. The overexpression of NLRP3 in N9 microglia by lentivirus reversed the effect of Gen pretreatment on the release of inflammatory factors and on neuronal injury. This demonstrated that NLRP3 inflammasome inhibition was necessary for Gen-induced protection. Therefore, we speculate that the NLRP3 inflammasome pathway in microglia might be the underlying mechanism of the beneficial effects of Gen.

Some limitations of this study need to be noted. Because the investigation was focused on the acute outcome of Gen in reproductively senescent mice, we only observed the first 24 h after ischemia, and no additional time points were considered. In addition, we tried to overexpress NLRP3 using a virus tool, however, neither the AAV nor the lentivirus could infect microglia *in vivo* or in primary microglia *in vitro*.

## Conclusion

By using the reproductively senescent mice in an *in vivo* MCAO model and an *in vitro* OGD model, our study shows that Gen attenuated the inflammatory response after ischemia by inhibiting the NLRP3 inflammasome in microglia within the acute phase of postmenopausal stroke, suggesting that Gen could be a promising neuroprotective agent for postmenopausal stroke.

## Data Availability Statement

The datasets generated for this study are available on request to the corresponding author.

## Ethics Statement

The animal study was reviewed and approved by Institutional Ethics Review Committee at Air Force Medical University.

## Author Contributions

SW and QY were responsible for experimental design, experiment conduction, data collection and analysis. SW and HW wrote the article. TG and JW helped in performing the study and participated in data collection. QY supervised the experiment and improved the manuscript. All authors approved the final manuscript.

## Conflict of Interest

The authors declare that the research was conducted in the absence of any commercial or financial relationships that could be construed as a potential conflict of interest.
