# Supplementary material for: Genistein Attenuates Acute Cerebral Ischemic Damage by Inhibiting the NLRP3 Inflammasome in Reproductively Senescent Mice
Source: Front Aging Neurosci. 2020 Jun 17;12:153. doi: 10.3389/fnagi.2020.00153 (PMC7311792; doi:10.3389/fnagi.2020.00153)
Supplement: Supplementary file 1 [file Table_1.doc]

**Supplementary Material**


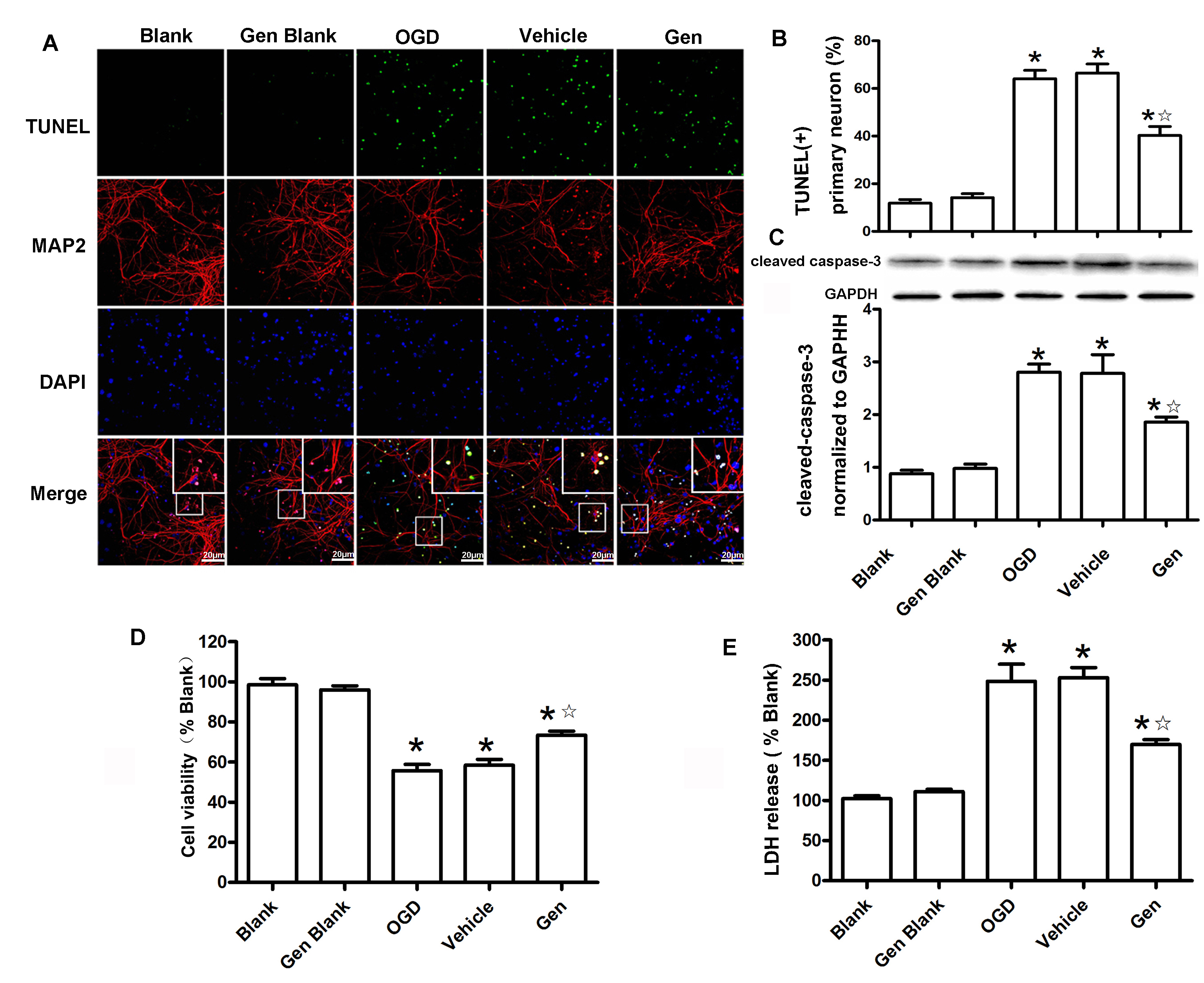
FIGURE S1

Microglia treated by Gen could reduce OGD/R primary neuronal injury in microglia-neuron coculture system. A, representative photomicrographs showing TUNEL staining of neurons in microglial-neuron co-culture system at 12 h after OGD/R. B, the percentage of TUNEL-positive cells in microglial-neuron co-cultured system. C, western blotting analysis of the cleaved-caspase-3 protein expression at 12 h after OGD/R. D, microglia treated by Gen significantly improved the cell viability of neurons. E, Gen treatment reduced LDH release. Data were presented as mean ± SD and analyzed by one-way ANOVA with Tukey post-test. **P* < 0.05 compared to Sham group. ^☆^*P* < 0.05 compared to Vehicle group, *n* = 4 per group.


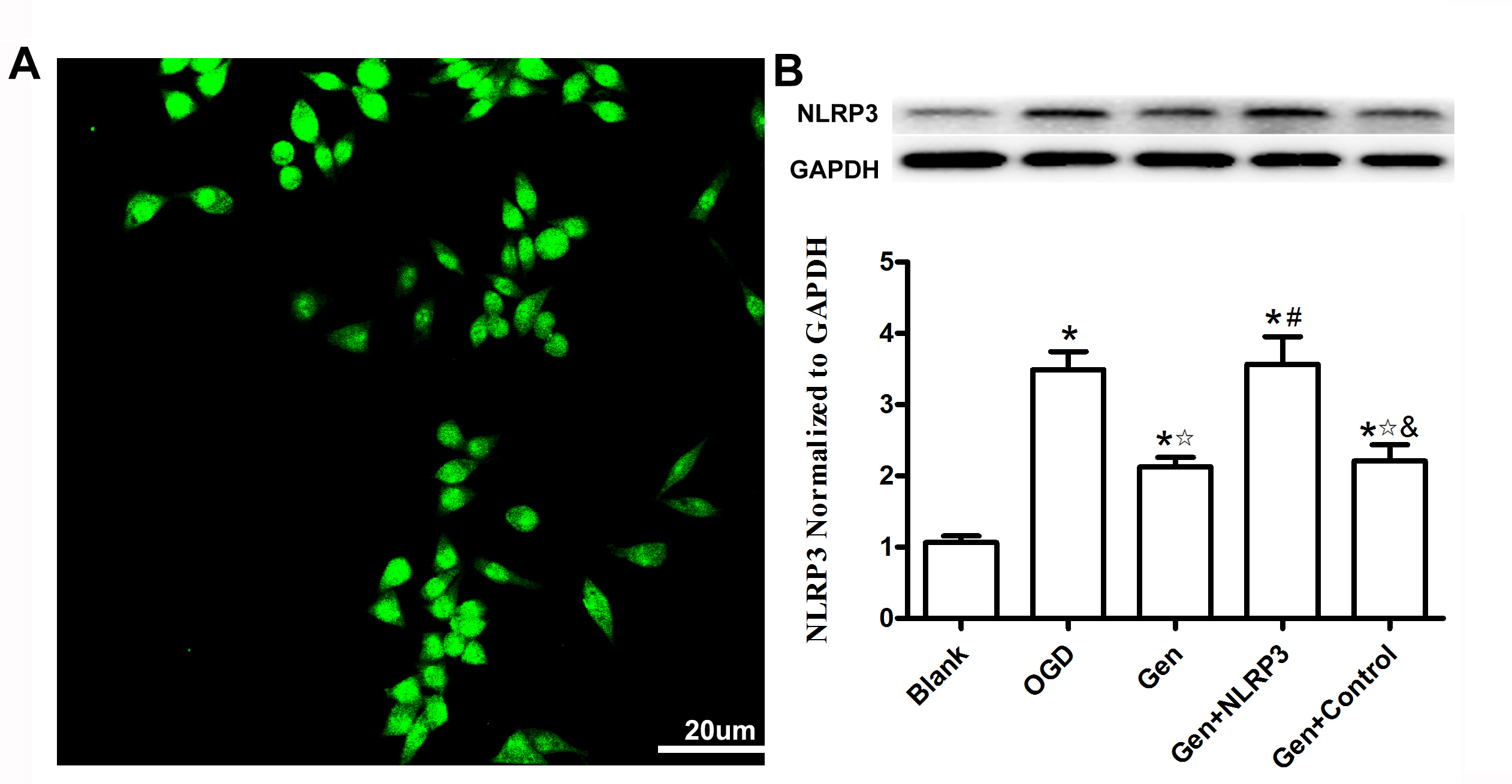


FIGURE S2

The verification of virus expression. A, the proportion of cells infected by the LV. B, NLRP3 expression in different groups of N9 cells. Data were presented as mean ± SD and analyzed by one-way ANOVA with Tukey post-test. **P* < 0.05 compared to Sham group, ^☆^*P* < 0.05 compared to OGD group, ^#^*P* < 0.05 compared to Gen group, ^&^*P* < 0.05 compared to Gen + NLRP3 group,  *n* = 4 per group.
